# The pharmacological bases for repurposing statins in depression: a review of mechanistic studies

**DOI:** 10.1038/s41398-023-02533-z

**Published:** 2023-07-12

**Authors:** Riccardo De Giorgi, Nicola Rizzo Pesci, Gianluca Rosso, Giuseppe Maina, Philip J. Cowen, Catherine J. Harmer

**Affiliations:** 1 University of Oxford, Department of Psychiatry, Warneford Hospital, Warneford Lane, Oxfordshire, Oxford, OX3 7JX United Kingdom; 2 Oxford Health NHS Foundation Trust, Warneford Hospital, Warneford Lane, Oxfordshire, Oxford, OX3 7JX United Kingdom; 3 University of Turin, Department of Neurosciences “Rita Levi Montalcini”, Via Cherasco 15, Turin, 10126 Italy

**Keywords:** Depression, Biomarkers, Pathogenesis, Clinical pharmacology, Molecular neuroscience

## Abstract

Statins are commonly prescribed medications widely investigated for their potential actions on the brain and mental health. Pre-clinical and clinical evidence suggests that statins may play a role in the treatment of depressive disorders, but only the latter has been systematically assessed. Thus, the physiopathological mechanisms underlying statins’ putative antidepressant or depressogenic effects have not been established. This review aims to gather available evidence from mechanistic studies to strengthen the pharmacological basis for repurposing statins in depression. We used a broad, well-validated search strategy over three major databases (Pubmed/MEDLINE, Embase, PsychINFO) to retrieve any mechanistic study investigating statins’ effects on depression. The systematic search yielded 8068 records, which were narrowed down to 77 relevant papers. The selected studies (some dealing with more than one bodily system) described several neuropsychopharmacological (44 studies), endocrine-metabolic (17 studies), cardiovascular (6 studies) and immunological (15 studies) mechanisms potentially contributing to the effects of statins on mood. Numerous articles highlighted the beneficial effect of statins on depression, particularly through positive actions on serotonergic neurotransmission, neurogenesis and neuroplasticity, hypothalamic-pituitary axis regulation and modulation of inflammation. The role of other mechanisms, especially the association between statins, lipid metabolism and worsening of depressive symptoms, appears more controversial. Overall, most mechanistic evidence supports an antidepressant activity for statins, likely mediated by a variety of intertwined processes involving several bodily systems. Further research in this area can benefit from measuring relevant biomarkers to inform the selection of patients most likely to respond to statins’ antidepressant effects while also improving our understanding of the physiopathological basis of depression.

## Introduction

Statins are among the most prescribed medications worldwide [1, 2]. Thanks to their established safety [3], statins are considered prototype candidates for ’drug repurposing’—an approach to find new therapeutic uses for well-known molecules; this approach can be useful in areas at high risk of failure in drug discovery such as psychiatry [4]. One strategy behind drug repurposing in psychiatry is based on the advances in our understanding of biological determinants of mental illness, which can then be targeted with molecules known to express the relevant pharmacological activity [4]. A classic example involves the repositioning of anti-inflammatory medications for the treatment of depression [5], which was initially promoted by the observation that depressive symptoms seen in chronic inflammatory disorders seem to respond to immune-active drugs regardless of concomitant physical health improvement [6]. Following this, a ’depressive-inflammatory’ subtype of depression has been increasingly established, and the same occurred for a variety of treatments aiming to benefit this subgroup of patients [7]. Among these, statins have been extensively investigated because of their recognised anti-inflammatory activity [8]. However, these medications also have several other molecular targets—primarily the reduction of cholesterol, that could argue against their use in depression: for example, previous data suggesting that low cholesterol, suicidality and low mood can be associated [9].

Overall, while statins’ general pharmacological actions are well-established, their broader effects—especially neuropsychopharmacological ones, are less clear and increasingly explored. The general pharmacology and neuropsychopharmacology of statins are now briefly summarised.

### General pharmacology of statins

Statins’ primary mechanism of action involves the competitive, reversible antagonism of liver 3-hydroxy-3-methylglutaryl-Coenzyme A (HMG-CoA) reductase, the rate-limiting enzyme in cholesterol biosynthesis [10]. By inhibiting HMG-CoA reductase, statins thwart the physiological production of cholesterol with a subsequent decline of its blood levels [11]. The ensuing reduction in cholesterol concentration within hepatocytes triggers the upregulation of low-density lipoprotein (LDL)-receptor via sterol regulatory element binding proteins [12], leading to increased uptake of LDL cholesterol from systemic circulation [13]. In other words, statins’ cholesterol-lowering properties depend not only on the reduction of cholesterol biosynthesis from the liver, but also on the consequent substantial upsurge in LDL clearance from plasma. Ancillary mechanisms of cholesterol reduction comprise inhibition of hepatic synthesis of apolipoprotein B100 [14] and decreased production and secretion of triglyceride-rich lipoproteins [15]. Overall, the effects on lipid profile include substantial contractions in total cholesterol, LDL, and triglycerides, as well as an accrue in high-density lipoproteins (HDLs) [16]. Additionally, statins differ from other lipid-lowering agents because their upstream inhibition of the mevalonate pathway affects several end-products other than cholesterol, which are responsible for numerous homoeostatic processes, including Coenzyme Q (mitochondrial respiratory chain), farnesyl- and geranyl-geranyl pyrophosphate moieties (protein post-translational modifications), isopentenyl tRNAs (RNA transcription), and dolichol (protein N-glycosylation) [17]. On this basis, statins are described as possessing ’pleiotropic effects’ [18].

### Neuropsychopharmacology of statins

There is increasing—though not definitive evidence that all statins, regardless of their lipophilicity, can reach the central nervous system (CNS) [19–22]. These molecules are detected in rodent brains after a few hours following a single dose administration [19]. Both lipophilic and hydrophilic statins can be found in the neuroparenchyma of animals [20] and humans [21], with functional magnetic resonance imaging (fMRI) studies displaying their effect on neural activity [22]. Consistent data indicate that statins can affect brain function both directly and indirectly [23, 24].

The local CNS effects of statins involve brain lipids, neurotransmission, neurogenesis and neuroprotection from trauma, inflammation, and oxidative stress [24]. Firstly, it should be noted that cholesterol and other end-products of the mevalonate pathway are especially abundant in the CNS, where they serve many essential physiological functions [25]. These molecules are rather metabolically inert in the adult brain: their half-life spans from months to years [26], and only some 0.02% undergo daily turnover through de novo synthesis mainly by astrocytes [27], meaning that there is no need to rely on uptake from systemic circulation [28]. Nevertheless, even short-term statins administration seems to cause acute disruption in the homoeostasis of these metabolites in the CNS [20], whereas chronic statin use determines further reductions in brain cholesterol [29] and other mevalonate end-products [30], either directly through HMG-CoA reductase inhibition, or secondarily via a ’sink effect’. Modulation of these lipids within the CNS leads to changes in brain function and behaviour, and is therefore associated with neuropsychological diseases [25] and their treatment [31].

Statins have widespread effects on neurotransmission, involving the monoaminergic, cholinergic and glutamatergic systems that have been implicated in a variety of neuropsychiatric disorders: both cholesterol-dependent and unrelated (e.g. anti-inflammatory and antioxidant) mechanisms can explain such alterations in neurotransmitters levels [32]. Statins are also ligands of peroxisome proliferator-activated receptor (PPAR)α, which drives the expression of neurotrophins such as brain-derived neurotrophic factor (BDNF) [33]. Furthermore, statin-dependent inhibition of the mevalonate pathway stimulates hippocampal neurogenesis via Wnt signalling [34] and promotes neurite outgrowth [35], though also appearing to inhibit synaptic spurring [36].

Finally, statins can be neuroprotective against a variety of stressors. Following traumatic injury, statin use is associated with reduced neuronal loss [37] and increased tissue recovery via vascular endothelial growth factor (VEGF) and activation of the PI3K/Akt-BDNF pathway [38]. Likewise, the suppression of certain mevalonate metabolites mediated by statins dampens the production of pro-inflammatory cytokines [39] and free radicals [40] such as reactive oxygen species (ROS) and nitric oxide (NO), thus protecting neurons from leaky blood-brain barrier (BBB) [41] and overly activated microglia [42] (i.e. neuroinflammation), as well as oxidative stress [43].

The peripheral effects of statins involve a wealth of systems, part of their dubbed ’pleiotropy’ [23]. In addition to, and independently from their established activity on the metabolism of bodily lipids [44, 45], statins can regulate critical functions of endocrine (e.g. cortisol [46, 47] and insulin secretion [48, 49]), cardiovascular (e.g. endothelial function, platelet activation and atherogenesis [50]), and immune (e.g. regulation of innate immunity via pro- and anti-inflammatory cytokines [51, 52] and of adaptive immunity via inhibition of antigen-1 leucocytes (LFA-1) [53], T-cell activation [54] and regulatory T-cells induction [55]) systems. All these processes share profound interactions with each other [56–59], not to mention their substantial crosstalk with the neurobiological mechanisms described above [23, 24].

### Aim of the review

Despite considerable research probing statins in a variety of neuropsychiatric disorders, and the growing amount of literature available on this topic, the effects of statins in neuropsychiatric disorders remain controversial [60]. Clinical studies show that statins are promising candidate molecules to repurpose in depression [61], but while evidence from trials and observational studies has been extensively summarised, both descriptively [62, 63] and quantitatively [64–72], the same cannot be said for mechanistic studies. A prior paper had described the neurobiological underpinnings potentially targeted by statins in mood disorders [73], but evidence had not been systematically drawn from studies that directly assessed statins’ use in depression—or models thereof.

The large amount of original research investigating the use of statins in depression, and the several articles attempting to summarise such evidence over the last few years, highlight that this is a topic of ongoing debate within the scientific community [62]. In this context, the design of further clinical research may benefit from a comprehensive overview of relevant translational findings.

Evidence from in vitro, animal, and human translational research is usually gathered and presented by means of narrative reviews. Because these studies are abundant yet less methodically organised on search engines and databases than their clinical counterparts, systematically searching for relevant mechanistic evidence can be daunting, though profitable [74]— and machine learning approaches have been developed to support the task [75]. In this paper, we, therefore, provide an overview of the mechanistic evidence that defines the pharmacological bases for repurposing statins in depression.

## Materials and methods

In this review, we used a broad search strategy conducted on three major databases (i.e. Pubmed/MEDLINE, Embase, PsychINFO) via OvidSP on 8 April 2022, updated then on 22 April 2023 following peer-review. The search algorithms combined index terms and free-text words for statins, depression or depressive symptoms, and depression-like models used in animals (Supplementary Material, S1). As advised for reviews of mechanistic studies, a web-based software (i.e. Rayyan) [76] for semi-automated text mining, and extensive forward/backward searching were employed to support de-duplicating and screening records. Two researchers (RDG, NRP) independently screened titles and abstracts for relevance, assessed the full texts for eligibility, and extracted relevant data. Disagreements were discussed with the other authors and resolved by *consensus*. Eventually, we only included mechanistic studies that reported original data on the pharmacological effects of statins in depression, with no restriction to their design and language.

## Results

**Fig. 1:**
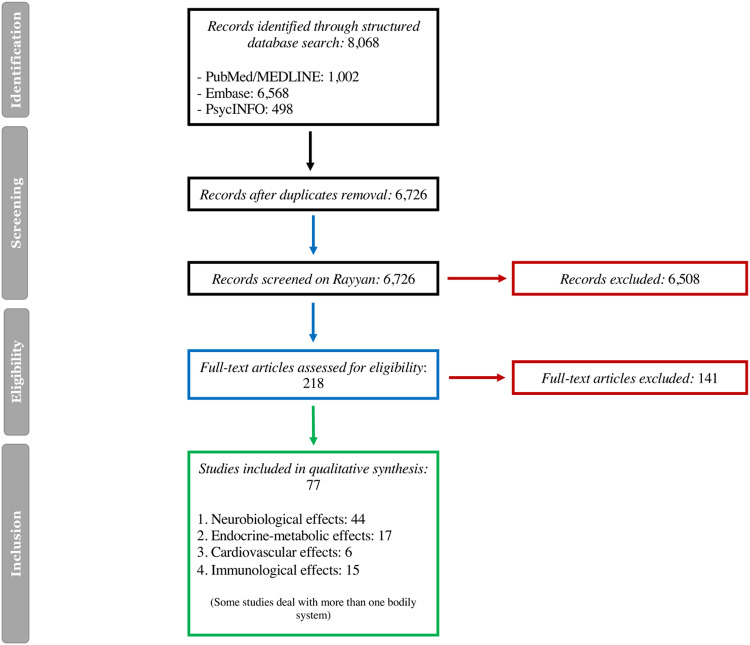
Flow chart of the search for mechanistic studies.

The search flow chart is in Fig. 1. The initial search yielded 6806 records, of which 2080 were duplicates. Screening of titles and abstracts led to the removal of 4548 non-relevant studies. Further 107 articles were excluded from the eligibility assessment of their full texts. Eventually, 77 studies were included in the review. Of these, the majority included animal models of depression (50 studies), six involved in vitro investigations and 21 were translational studies in human participants.

**Fig. 2:**
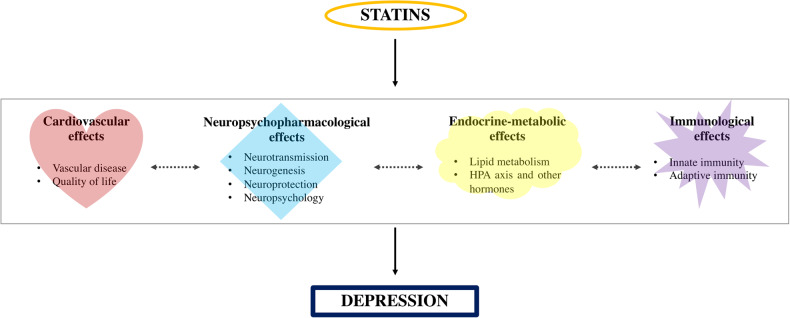
Current mechanisms explaining the effects of statins in depression.

Overall, mechanistic evidence showed that several intertwined neuropsychopharmacological (44 studies), endocrine-metabolic (17 studies), cardiovascular (6 studies) and immunological (15 studies) processes may contribute to the effects of statins in depression (Fig. 2).

Each included study was described in its relevant section(s) and summarised in Table 1 (see also Supplementary Material, S2).

**Table 1:**
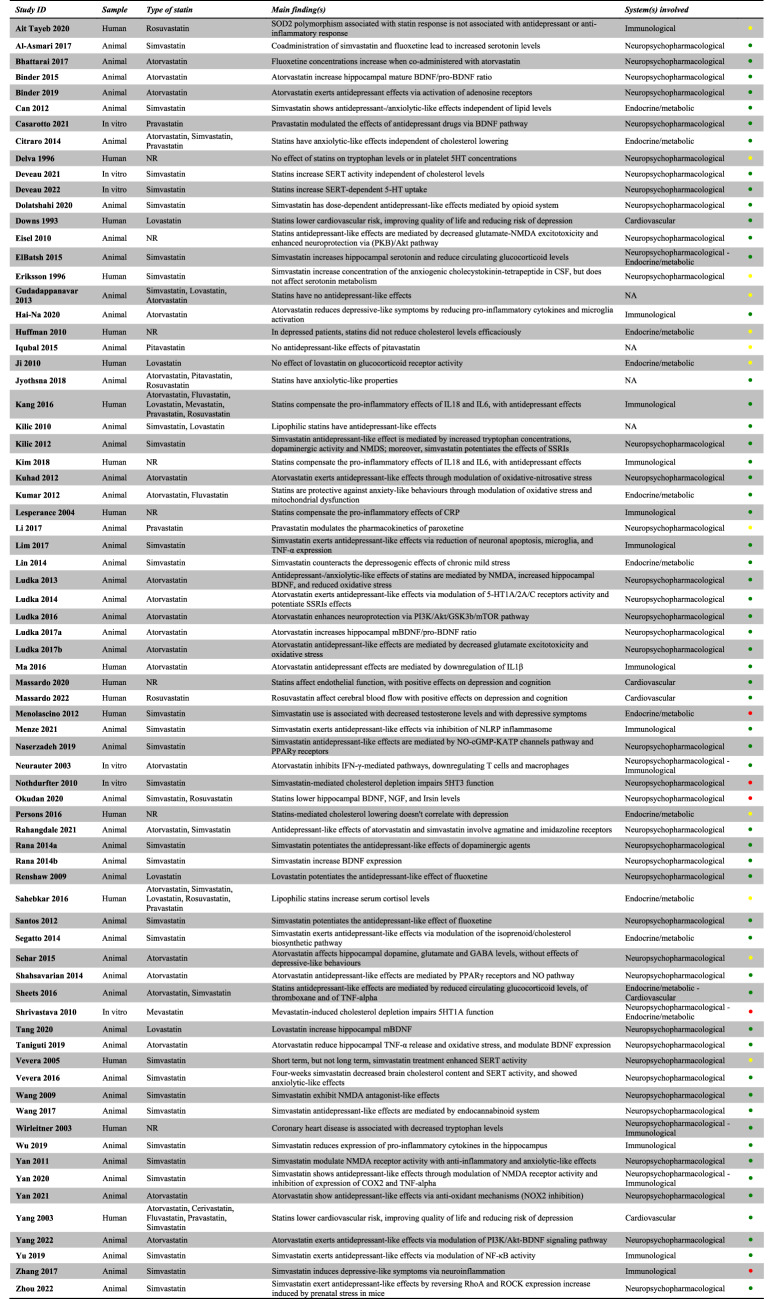
Summary of included studies.

Green: positive effect; yellow: no effect; red: negative effect.

Further four studies assessed the putative antidepressant activity of statins in animal models of depression without further investigating their underlying mechanism of action and are here briefly reported. These showed that the highly-lipophilic simvastatin and lovastatin have antidepressant-like effects in rats or mice [77], while the less lipophilic atorvastatin [78] and pitavastatin [79] fail to do so. However, atorvastatin, pitavastatin and hydrophilic rosuvastatin display antianxiety properties [80].

### Neuropsychopharmacological effects of statins in depression

These include effects on neurotransmission, neurogenesis, neuroprotection and neuropsychology.

#### Neurotransmission

The pathophysiology of depression is classically associated with anomalies in monoaminergic (i.e. serotonin or 5-hydroxytryptamine, 5HT; noradrenaline, NA; dopamine, DA) neurotransmission [81], though more recently glutamatergic, γ-aminobutyric acid (GABA)ergic, and cholinergic receptors have been implicated [82]. Numerous studies indicate that statins can alter synaptic transmission by modulating the function of several of these neurotransmitter receptors and their ligands [32].

##### Serotonin

In vitro, statin-induced cholesterol depletion impairs 5HT_1A_ [83] and 5HT_3_ [84] receptor function. Simvastatin also increases serotonin reuptake by augmenting serotonin transporter (SERT) activity via both cholesterol-mediated [85] and independent [86] pathways. These effects would apparently decrease serotonin activity. Nevertheless, animal models have shown an antidepressant-like effect of simvastatin which may be linked to an increase in the availability of tryptophan, the serotonin precursor, through the inhibition of indoleamine 2,3-dioxygenase (IDO) [87], and increases in hippocampal serotonin [88], as well as reduced SERT activity [89]. Conversely, serotonin depletion or 5HT_1A_ and 5HT_2A/C_ receptor antagonism abolish the antidepressant effect of atorvastatin [90]. These findings have not been replicated in human studies assessing 5HT neuroendocrine function and plasma tryptophan in hypercholesterolaemic patients receiving statins [91]. Furthermore, simvastatin appeared to increase SERT function in the short-, but not long-term in humans [92].

Statins can also modulate the serotonergic effects of some antidepressants in vitro, via the tyrosine kinase receptor 2 (TRKB) domain of BDNF receptor [93]. In animals, the antidepressant effect of selective serotonin reuptake inhibitors (SSRIs) seems potentiated by several statins [87, 90, 94, 95], possibly involving pharmacokinetics interactions [96–98], but the same does not apply to tricyclic antidepressants (TCAs) [87].

##### Dopamine

Because dopamine neural circuitry, difficult-to-treat depressive symptoms (especially anhedonia), and inflammation appear reliably related [99, 100], statins might be ideally placed to modulate these mechanisms at the same time. Indeed, the dopaminergic system appears affected by statins administration, but while certain studies demonstrate the occurrence of concomitant dopaminergic and antidepressant- or anxiolytic-like effects [87], perhaps mediated by interaction with BDNF function [101, 102] or via potentiation of dopaminergic mechanisms [103] for simvastatin, others fail to show any concurrent changes in animal depressive or anxiety behaviour for atorvastatin [104].

##### Glutamate and GABA

The most recent and successful developments in depression therapeutics have not been confined to monoaminergic systems but have focussed instead on molecules capable of targeting the glutamatergic and GABAergic pathways [105](e.g., ketamine and esketamine for depression and suicidality [106], brexanolone for post-partum depression [107]). The antidepressant and anxiolytic properties of simvastatin [87, 108–111] and atorvastatin [112] in rats seem linked to glutamate N-methyl-D-aspartate (NMDA) receptor expression and blockade, especially in the hippocampus and amygdala. However, another study showed that while atorvastatin seems to affect hippocampal glutamate and GABA, no concurrent effect on depression or anxiety can be observed in mice [104].

##### Other neurotransmitters

Less conventional pathways have also been explored in animal models of depression, showing that simvastatin may elicit antidepressant-like action via opioid- [113] and endocannabinoid-mediated [114] neurotransmission, while atorvastatin does so via adenosine-dependant pathways [115]. Simvastatin might also increase the concentration of the anxiogenic cholecystokinin-tetrapeptide in the cerebrospinal fluid (CSF) of healthy human subjects [116] while no effect on CSF serotonin or its metabolite 5-HIAA was found.

#### Neurogenesis

Processes of hippocampal neurogenesis and neuroplasticity, largely controlled by neurotrophins such as BDNF [117], are considered today a hallmark of depressive disorder and antidepressant action [118].

Emerging evidence from animal studies suggests that lovastatin and atorvastatin may enhance the proteolytic cleavage of pro-BDNF [119–121], BDNF hippocampal concentrations [112, 122] and α7nAChR-mediated activation of the PI3K/Akt-BDNF pathway [123], with a consequent positive influence on depressive-like behaviour. Agmatine and imidazoline receptors, whose function broadly relates to BDNF neurogenesis, NMDA neuroprotection and monoamine regulation, have also been involved in the antidepressant-like effect of simvastatin and atorvastatin [124]. On the other hand, simvastatin or rosuvastatin administration seem associated with lower hippocampal BDNF and anxiogenic response in rats [125].

#### Neuroprotection

Excitotoxicity [126] and oxidative stress due to reactive oxygen and nitrogen species in the brain [127] are strictly related, to highly depressogenic triggers.

Numerous animal studies show that the antidepressant effect of statins may occur because of decreasing glutamate-NMDA excitotoxicity [128], PPARγ-mediated [129, 130] or inflammation-related [131, 132] nitrosative and oxidative stress, or all the above [112, 133], while also inducing neuroprotective pathways such as protein kinase B (PKB)/Akt [128], PI3K/Akt-GSK3b/mTOR [134] and RhoA/ROCK [135] signalling.

#### Neuropsychology

The cognitive neuropsychological model of depression uses changes in emotional processing as a biomarker for depressive disorders and the assessment of antidepressant or depressogenic responses [136]. Negative bias in emotional processing has long been recognised as a core feature of depression, leading to a vicious circle of negative feelings, thoughts and behaviour which triggers and maintains depressive symptoms [137]. These emotional biases can occur across several cognitive domains, including perception, attention and memory [138]: for example, people with depression are more likely to perceive and categorise facial expressions as negative or to attend and recall negative information in emotional word-based tasks [139].

The cognitive effects of statins have been investigated for several years [140], but only a few, recent studies have done so in humans in the context of depression. Firstly, an observational study shows a favourable association between statins use and lower recognition of negative faces, with increased misclassification of these expressions as positive, predicting increased depression and anxiety symptoms at later assessments [141]. Conversely, two experimental medicine trials respectively find that atorvastatin [142] and simvastatin [143] have negative or no effects on emotional processing.

### Endocrine-metabolic effects of statins in depression

These include effects on lipid metabolism and on the hypothalamic-pituitary-adrenal (HPA) axis and other hormones.

#### Lipid metabolism

Lipids in the CNS and peripheral circulation interact with biological pathways implicated in depression [144] and antidepressant action [145]. Intriguingly, lipid homoeostasis is critical to several interconnected mechanisms involved in mood regulation, anxiety and suicidal behaviour, including serotonin neurotransmission [146–148], neurogenesis [149], neuroprotection from excitotoxicity [109] and systemic inflammation [148]. From a clinical standpoint, dyslipidaemia and depression, its severity and prospective course appear associated [150], while SSRI-induced increase in cholesterol has been argued to be protective against depression [151]. Correlations between depressive symptomatology and both raised [152, 153] and diminished [154–157] concentrations of circulating lipids, including total cholesterol, LDL, HDL, triglycerides, ω-3 polyunsaturated fatty acids (PUFA), can be observed. These associations may differ between men and women [158]. Some studies highlight a link between cholesterol and anxiety, rather than an effect on mood [159]. Nevertheless, changes in lipid metabolism have been proposed as potential biomarkers for depressive disorders [160].

In keeping with the variable findings above, the effects of statins on lipid metabolism and thus depression seem conflicting. An in vitro study shows that statin-mediated cholesterol depletion inhibits 5HT_1A_ receptor dynamics [83]. High-fat diet induces depressive and anxiety behaviours in rats, but these effects are counteracted by simvastatin [161]. Also, simvastatin administration affects mevalonate metabolites within the hippocampus and prefrontal cortex of rodents, with consequent modulation of emotional cognition [17], though another study highlights a detrimental association between cholesterol-lowering and altered behaviour, weight loss, and circadian disruption [162]. On the other hand, the antidepressant- and anxiolytic-like effects of several statins are observed in rats in the absence of concurrent changes in plasma cholesterol [163]. An intriguing study in humans reports that, despite low LDL cholesterol levels correlating with depression, as also described above, such association is not observed when cholesterol-lowering is achieved via statins [164]. Furthermore, failure to improve the lipid profile, following statin therapy, in patients who suffered a myocardial infarction, seems associated with a higher incidence of depression at 6 months [165].

#### Hypothalamic-pituitary-adrenal axis and other hormones

Disturbances of glucocorticoids and the HPA or ’stress’ axis, closely related to abnormal inflammatory response, are known to play a major role in the pathophysiology of depression [166], with elevated plasmatic cortisol potentially predicting the development of depressive disorders [167].

Some reviews have hypothesised that statins may mediate the relationship between lipid metabolism, stress, inflammation, and depression in animals [168] and humans [169, 170]. Simvastatin [88] and atorvastatin [171] reduce glucocorticoid levels while expressing antidepressant-like effects in rats. Equally, the depressive- [172] and anxiety-like [173] behaviours caused by chronic mild stress are neutralised with statins use. In humans however statins have been observed to have no effect on glucocorticoid receptors [174] or to even increase serum cortisol [46], although neither study specifically addresses whether these events eventually lead to the development of depression. A case report instead describes the onset of depressive symptomatology in a male whose simvastatin initiation was associated with a reduction of testosterone levels [175].

### Cardiovascular effects of statins in depression

These include effects on vascular disease and overall quality of life.

#### Vascular disease

Atherosclerosis and endothelial dysfunction, which statins lessen via both cholesterol-mediated and other mevalonate-dependant pathways, appear involved in depression [176, 177], especially in late-life according to the ’vascular depression hypothesis’ [178]. A recent meta-analysis has indeed identified a pattern of increased hyperintensity burden on magnetic resonance imaging (MRI) in people whose depression has a late onset [179]. Furthermore, there is a clear bidirectional association between depression and cardiovascular morbidity and mortality [180, 181], therefore interventions that are capable of targeting both mechanisms could yield particular benefit.

Statins are considered excellent candidates for reducing vascular dysfunction of the small white matter vessels in the neuroparenchyma, with consequent positive effects on depression [182]. In obese rats, atorvastatin administration reduces thromboxane and improves vascular reactivity while decreasing depressive-like behaviour [171]. One recent human study shows that low doses of statins in depressed participants determine blood flow changes in key brain areas of mood and cognitive control as well as an improvement in depressive symptoms and markers of endothelial function [183, 184].

#### Quality of life

On the back of strong bidirectional links between depression, cardiovascular disorders and quality of life [185], some authors argue the ability of statins to prevent cardiovascular and cerebrovascular accidents can lead to improved quality of life and thus lower onset of depressive disorders [186, 187]. However, no studies that explicitly investigate this issue in humans could be retrieved.

### Immunological effects of statins in depression

These include effects on innate immunity or inflammation and adaptive immunity.

#### Innate immunity (inflammation)

Extensive evidence suggests that immune processes, especially inflammatory ones, are prominent in depression pathophysiology [7]. Both peripheral and CNS inflammation appear causally involved [188].

Simvastatin [189–193] and atorvastatin [194] reduce depressive-like symptoms in animals by decreasing neuroinflammation thanks to the suppression of pro-inflammatory cytokines, P2X7-inflammasome complex, and microglia activation. In addition, the reduction of circulating tumour necrosis factor (TNF)α by simvastatin [171] and atorvastatin [195] is likewise associated with improved depressive-like behaviour. Some translational human studies indicate that statins might positively affect mood by offsetting the peripheral pro-inflammatory effects of interleukin (IL)1β [196], IL6 and IL18 [197, 198] and C-reactive protein (CRP) [199]. Nonetheless, a study on a functional genetic polymorphism of superoxide dismutase (SOD)2, an enzyme responsible for the anti-inflammatory activity of rosuvastatin, could not observe any association with an antidepressant response or CRP [200].

#### Adaptive immunity

Though with less consistency, disruptions in adaptive immunity (i.e. acquired humoral and cell-mediated immune system) have been implicated in depression [201].

No studies could be identified that directly assessed the effect of statins on these mechanisms. One study shows that atorvastatin can inhibit interferon (IFN)γ-dependant cellular immunity, which is related to increased tryptophan availability [202]. Since tryptophan is the precursor of serotonin, it is suggested that statins might reduce the risk of depression by decreasing immune-mediated tryptophan degradation [203, 204].

## Discussion

In this article, after recapitulating the general pharmacological and neuropsychopharmacological activities of statins, we reviewed the mechanistic evidence for the effects of these drugs in depression. While a few studies only assessed the behavioural consequences of statins administration in animal models of depression, the great majority (67 studies) of the investigations were mechanistic in nature, thus providing valuable insights on the interactions between statin use, depressive and anxiety symptoms, and numerous biological and psychological mechanisms.

Overall, most studies pointed toward an antidepressant and anxiolytic effect of statins by means of neurobiological, endocrine-metabolic, cardiovascular, and immunological mechanisms largely communicating with each other. A minority of investigations reported no effect, or even depressogenic and anxiogenic ones. Among the few in vitro studies, most identified a modulatory role of statins on serotoninergic pathways, possibly supporting some clinical evidence that statins’ effects in depression might be related to their ability to augment traditional antidepressants [64]. Evidence from the numerous studies in animal models of depression appears particularly suggestive of statins’ benefit: 32/36 studies showed a positive effect by influencing neurotransmitters turnover, neuroreceptors function, and neuroplasticity (two studies showed no effect [98, 104] and one a negative effect [125]), 7/7 studies via lipid metabolism and HPA axis regulation, and 7/8 studies via modulation of circulating molecules involved in immunological and cardiovascular function (one study showed, however, an increase in neuroinflammatory markers [190]). Findings from human translational studies were instead mixed: 10/201studies identified a potentially beneficial effect mainly mediated by anti-inflammatory and cardioprotective mechanisms, while the remaining showed either no effect or indeed a negative one on neurobiological, neuropsychological, and endocrine-metabolic processes – the latter perhaps in keeping with well-documented literature about the associations between low levels of cholesterol and some depressive symptoms [205]. Nevertheless, negative pre-clinical findings are less frequently published [206], therefore the dearth of the latter associations might not reflect a lack of harmful effects for statins. It is also important to notice that several other bodily systems probably affected by statins administration, such as the gut-brain axis [207], have not been assessed in the context of depression yet, and warrant further investigation.

Meanwhile, a few new clinical studies have recently been completed [208] or are ongoing (NCT04301271, NCT04685642), which may provide important insights not only on establishing the clinical efficacy of statins in depression, but also on mechanistic aspects of such effects (or lack thereof). Specifically, the last published clinical trial [208] has investigated the putative antidepressant effect of adjunctive (i.e., in addition to standard care) simvastatin in a large sample of 150 adults with treatment-resistant depression followed up for 12 weeks. This study design includes several features (e.g., use of the most lipophilic simvastatin, focus on a subgroup of patients with treatment-resistant depression, measurement of baseline lipid and inflammatory markers) that both pre-clinical and clinical evidence would support [61]—which is why the lack of any beneficial effect of statin compared to placebo, regardless of the mediating effect of lipid and inflammatory markers, appears disappointing [208] in contrast with earlier promising, yet smaller trials [64, 68]. Nevertheless, a large amount of clinical evidence (extensively reviewed elsewhere [63], see also Supplementary Material, S3 for an up-to-date list of studies) continues supporting the value of identifying subgroups of patients whose specific depression phenotype (as based on neuropsychopharmacological, endocrine-metabolic, cardiovascular, immunological or other markers) may be more responsive to, or preventable with, targeted statin treatment [208].

### Limitations

This review has several limitations. Although we used a broad and systematised approach to literature searching, it is possible that some records may have been missed, especially from grey literature, because pre-clinical studies are generally much more numerous and less methodically organised in databases than their clinical counterparts [209]. Overall, our work remains a narrative overview of mechanistic evidence, which includes a variety of heterogeneous studies including in vitro, animal, and human (both in clinically depressed and healthy populations) investigations. As such, the review was not been pre-registered, there was no attempt at pooling results to produce new evidence, and we did not systematically assess for sources of bias in the studies included—though we followed available advice on narrative reviews reporting [210] (Supplementary Material, S4). In this context, it should be noted that the internal validity of many pre-clinical experiments is sometimes poor, while publication bias is common [74]—meaning that caution is required when drawing any conclusion from the evidence reported.

## Conclusion

The translation of findings from in vitro, animal, and indeed human studies to medical practice remains a particular challenge for mental illnesses [211]. Consequently, the repurposing of medications based on the targeting of molecular pathways shown to be associated with the course of psychiatric diseases [4], such as mood disorders, has thus far produced modest results [212]. Mechanistic reasoning or “pathophysiologic rationale”—as compared to evidence produced via clinical trials, has often led to unjustified interpretations, to the extent that most evidence-based medicine proponents are legitimately sceptical about using such reasoning as evidence for efficacy or harm [213]. Nevertheless, the design of further pre-clinical and clinical studies investigating the effects of statins—or of any molecule targeting the physiopathological pathways examined above, as well as measurement of related biomarkers for depression and antidepressant response, may be informed by the evidence presented in this review.

### Supplementary information

Supplementary material
