# Supplementary material for: The pharmacological bases for repurposing statins in depression: a review of mechanistic studies
Source: Transl Psychiatry. 2023 Jul 12;13:253. doi: 10.1038/s41398-023-02533-z (PMC10338465; doi:10.1038/s41398-023-02533-z)
Supplement: Supplementary file 1 — Supplementary material [file 41398_2023_2533_MOESM1_ESM.docx]

**SUPPLEMENTARY MATERIAL,**

## S1 – Search strategy

#### MEDLINE Ovid SP

#1 exp Depressive disorder/

#2 Depression/

#3 (depress$ OR dysthymi$ OR mood OR affectiv$).ti,ab,kf

#4 (reserpin$ OR learned helplessness OR chronic mild stress OR social defeat stress OR forced swimming OR tail suspension OR open field OR sucrose preference OR novelty suppressed feeding OR elevated plus maze).ti,ab,kf

#5 or/1-4

#6 exp Hydroxymethylglutaryl-CoA Reductase Inhibitors/

#7 (statin$ OR atorvastatin OR cerivastatin OR fluvastatin OR lovastatin OR pitavastatin OR pravastatin OR rosuvastatin OR simvastatin).tw

#8 or/6-7

#9 5 AND 8

#### Embase Ovid SP

#1 exp Depression/

#2 (depress$ OR dysthymi$ OR mood OR affectiv$).ti,ab,kw

#3 (reserpin$ OR learned helplessness OR chronic mild stress OR social defeat stress OR forced swimming OR tail suspension OR open field OR sucrose preference OR novelty suppressed feeding OR elevated plus maze).ti,ab,kw

#4 or/1-3

#5 exp hydroxymethylglutaryl coenzyme A reductase inhibitor/

#6 (statin$ OR atorvastatin OR cerivastatin OR fluvastatin OR lovastatin OR pitavastatin OR pravastatin OR rosuvastatin OR simvastatin).tw

#7 or/5-6

#8 4 AND 7

#### PsycINFO Ovid SP

#1 depression.hw

#2 (depress$ OR dysthymi$ OR mood OR affectiv$).ti,ab,id

#3 (reserpin$ OR learned helplessness OR chronic mild stress OR social defeat stress OR forced swimming OR tail suspension OR open field OR sucrose preference OR novelty suppressed feeding OR elevated plus maze).ti,ab,id

#4 or/1-3

#5 exp statins/

#6 (statin$ OR atorvastatin OR cerivastatin OR fluvastatin OR lovastatin OR pitavastatin OR pravastatin OR rosuvastatin OR simvastatin).tw

#7 or/5-6

#8 4 AND 7

## S2 – Table 1, list of included studies by mechanism

| *Study ID* | *Sample* | *Type of statin* | *Main finding(s)* | | | |  |  |  |
| --- | --- | --- | --- | --- | --- | --- | --- | --- | --- |
| Neuropsychopharmacological effects | | | | | | |  |  |  |
| Al-Asmari 2017 | Animal | Simvastatin | Coadministration of simvastatin and fluoxetine lead to increased serotonin levels | | | | • |  |  |
| Bhattarai 2017 | Animal | Atorvastatin | Fluoxetine concentrations increase when co-administered with atorvastatin | | | | • |  |  |
| Binder 2015 | Animal | Atorvastatin | Atorvastatin increase hippocampal mature BDNF/pro-BDNF ratio | | | | • |  |  |
| Binder 2019 | Animal | Atorvastatin | | Atorvastatin exerts anti-depressant effects via activation of adenosine receptors | | | • |  |  |
| Casarotto 2021 | In vitro | Pravastatin | Pravastatin modulated the effects of antidepressant drugs via BDNF pathway | | | | • |  |  |
| Delva 1996 | Human | NR | No effect of statins on tryptophan levels or in platelet 5HT concentrations | | | | • |  |  |
| Deveau 2021 | In vitro | Simvastatin | Statins increase SERT activity independent of cholesterol levels | | | | • |  |  |
| Deveau 2022 | In vitro | Simvastatin | Statins increase SERT-dependent 5-HT uptake | | | | • |  |  |
| Dolatshahi 2020 | Animal | Simvastatin | Simvastatin has dose-dependent antidepressant-like effects mediated by opioid system | | | | • |  |  |
| Eisel 2010 | Animal | NR | Statins antidepressant-like effects are mediated by decreased glutamate-NMDA excitotoxicity and enhanced neuroprotection via (PKB)/Akt pathway | | | | • |  |  |
| ElBatsh 2015 | Animal | Simvastatin | Simvastatin increase hippocampal serotonin and reduce circulating glucocorticoid levels | | | | • |  |  |
| Eriksson 1996 | Human | Simvastatin | Simvastatin increase concentration of the anxiogenic cholecystokinin-tetrapeptide in CSF, but does not affect serotonin metabolism | | | | • |  |  |
| Kilic 2012 | Animal | Simvastatin | Simvastatin antidepressant-like effect is mediated by increased tryptophan concentrations, dopaminergic activity and NMDS; moreover, simvastatin potentiates the effects of SSRIs | | | | • |  |  |
| Kuhad 2012 | Animal | Atorvastatin | Atorvastatin exert antidepressant-like effects through modulation of oxidative-nitrosative stress | | | | • |  |  |
| Li 2017 | Animal | Pravastatin | Pravastatin modulates the pharmacokinetics of paroxetine | | | | • |  |  |
| Ludka 2013 | Animal | Atorvastatin | Antidepressant-/anxiolytic-like effects of statins are mediated by NMDA, increased hippocampal BDNF, and reduced oxidative stress | | | | • |  |  |
| Ludka 2014 | Animal | Atorvastatin | Atorvastatin exerts antidepressant-like effects via modulation of 5-HT1A/2A/C receptors activity and potentiate SSRIs effects | | | | • |  |  |
| Ludka 2016 | Animal | Atorvastatin | Atorvastatin enhance neuroprotection via PI3K/Akt/GSK3b/mTOR pathway | | | | • |  |  |
| Ludka 2017a | Animal | Atorvastatin | Atorvastatin increases hippocampal mBDNF/pro-BDNF ratio | | | | • |  |  |
| Ludka 2017b | Animal | Atorvastatin | Atorvastatin antidepressant-like effects are mediated by decreased glutamate excitotoxicity and oxidative stress | | | | • |  |  |
| Naserzadeh 2019 | Animal | Simvastatin | Simvastatin antidepressant-like effects are mediated by NO-cGMP-KATP channels pathway and PPARγ receptors | | | | • |  |  |
| Neurauter 2003 | In vitro | Atorvastatin | Atorvastatin inhibits IFN-γ-mediated pathways, downregulating T cells and macrophages | | | | • |  |  |
| Nothdurfter 2010 | In vitro | Simvastatin | Simvastatin-mediated cholesterol depletion impairs 5HT3 function | | | | • |  |  |
| Okudan 2020 | Animal | Simvastatin, Rosuvastatin | Statins lower hippocampal BDNF, NGF and Irisin levels | | | | • |  |  |
| Rahangdale 2021 | Animal | Atorvastatin, Simvastatin | Antidepressant-like effects of atorvastatin and simvastatin involve agmatine and imidazoline receptors | | | | • |  |  |
| Rana 2014a | Animal | Simvastatin | Simvastatin potentiate the antidepressant-like effects of dopaminergic agents | | | | • |  |  |
| Rana 2014b | Animal | Simvastatin | Simvastatin increase BDNF expression | | | | • |  |  |
| Renshaw 2009 | Animal | Lovastatin | Lovastatin potentiate the antidepressant-like effect of fluoxetine | | | | • |  |  |
| Santos 2012 | Animal | Simvastatin | Simvastatin potentiate the antidepressant-like effect of fluoxetine | | | | • |  |  |
| Sehar 2015 | Animal | Atorvastatin | Atorvastatin affect hippocampal dopamine, glutamate and GABA levels, without effects of depressive-like behaviours | | | | • |  |  |
| Shahsavarian 2014 | Animal | Atorvastatin | Atorvastatin antidepressant-like effects are mediated by PPARγ receptors and NO pathway | | | | • |  |  |
| Shrivastava 2010 | In vitro | Mevastatin | Mevastatin-induced cholesterol depletion impairs 5HT1A function | | | | • |  |  |
| Tang 2020 | Animal | Lovastatin | Lovastatin increase hippocampal mBDNF | | | | • |  |  |
| Taniguti 2019 | Animal | Atorvastatin | Atorvastatin reduce hippocampal TNF-α release and oxidative stress, and modulate BDNF expression | | | | • |  |  |
| Vevera 2005 | Human | Simvastatin | | Short term, but not long term, simvastatin treatment enhanced SERT activity | | • | |  |  |
| Vevera 2016 | Animal | Simvastatin | | Four-weeks simvastatin decreased brain cholesterol content and SERT activity, and showed anxiolytic-like effects | | • | |  |  |
| Wang 2009 | Animal | Simvastatin | Simvastatin exhibit NMDA antagonist-like effects | | | | • |  |  |
| Wang 2017 | Animal | Simvastatin | Simvastatin antidepressant-like effects are mediated by endocannabinoid system | | | | • |  |  |
| Wirleitner 2003 | Human | NR | Coronary heart disease is associated with decreased tryptophan levels | | | | • |  |  |
| Yan 2011 | Animal | Simvastatin | Simvastatin modulate NMDA receptor activity with anti-inflammatory and anxiolytic-like effects | | | | • |  |  |
| Yan 2020 | Animal | Simvastatin | | Simvastatin shows antidepressant-like effects through modulation of NMDA receptor activity and inhibition of expression of COX2 and TNF-alpha | • | | |  |  |
| Yan 2021 | Animal | Atorvastatin | | Atorvastatin show antidepressant-like effects via anti-oxidant mechanisms (NOX2 inhibition) | • | | |  |  |
| Yang 2022 | Animal | Atorvastatin | Atorvastatin exerts antidepressant-like effects via modulation of PI3K/Akt-BDNF signalling pathway | | | | • |  |  |
| Zhou 2022 | Animal | Simvastatin | | Simvastatin exert antidepressant-like effects by reversing RhoA and ROCK expression increase induced by prenatal stress in mice |  |  |  |  |  |
| Endocrine-metabolic effects | | | | | | |  |  |  |
| Can 2012 | Animal | Simvastatin | Simvastatin show antidepressant-/anxiolytic-like effects independent of lipid levels | | | | • |  |  |
| Citraro 2014 | Animal | Atorvastatin, Simvastatin, Pravastatin | Statins have anxiolytic-like effects independent of cholesterol lowering | | | | • |  |  |
| ElBatsh 2015 | Animal | Simvastatin | Simvastatin increase hippocampal serotonin and reduce circulating glucocorticoid levels | | | | • |  |  |
| Huffman 2010 | Human | NR | In depressed patients statins did not reduce cholesterol levels efficaciously | | | | • |  |  |
| Ji 2010 | Human | Lovastatin | No effect of lovastatin on glucocorticoid receptor activity | | | | • |  |  |
| Kumar 2012 | Animal | Atorvastatin, Fluvastatin | Statins are protective against anxiety-like behaviours through modulation of oxidative stress and mitochondrial dysfunction | | | | • |  |  |
| Lin 2014 | Animal | Simvastatin | Simvastatin counteracts the depressogenic effects of chronic mild stress | | | | • |  |  |
| Menolascino 2012 | Human | Simvastatin | Simvastatin use is associated with decreased testosterone levels and with depressive symptoms | | | | • |  |  |
| Persons 2016 | Human | NR | Statins-mediated cholesterol lowering doesn't correlate with depression | | | | • |  |  |
| Sahebkar 2016 | Human | Atorvastatin, Simvastatin, Lovastatin, Rosuvastatin, Pravastatin | Lipophilic statins increase serum cortisol levels | | | | • |  |  |
| Segatto 2014 | Animal | Simvastatin | Simvastatin exerts antidepressant-like effects via modulation of the isoprenoid/cholesterol biosynthetic pathway | | | | • |  |  |
| Sheets 2016 | Animal | Atorvastatin, Simvastatin | Statins antidepressant-like effects are mediated by reduced circulating glucocorticoid levels, of thromboxane and of TNF-alpha | | | | • |  |  |
| Shrivastava 2010 | In vitro | Mevastatin | Mevastatin-induced cholesterol depletion impairs 5HT1A function | | | | • |  |  |
| Vevera 2016 | Animal | Simvastatin | | Four-weeks simvastatin decreased brain cholesterol content and serotonin transporter activity, and showed anxiolytic-like effects | | | • |  |  |
| Cardiovascular effects | | | | | | |  |  |  |
| Downs 1993 | Human | Lovastatin | Statins lower cardiovascular risk, improving quality of life and reducing risk of depression | | | | • |  |  |
| Massardo 2020 | Human | NR | Statins affect endothelial function, with positive effects on depression and cognition | | | | • |  |  |
| Massardo 2022 | Human | Rosuvastatin | Rosuvastatin affect cerebral blood flow with positive effects on depression and cognition | | | | • |  |  |
| Sheets 2016 | Animal | Atorvastatin, Simvastatin | Statins antidepressant-like effects are mediated by reduced circulating glucocorticoid levels, of thromboxane and of TNF-alpha | | | | • |  |  |
| Yang 2003 | Human | Atorvastatin, Cerivastatin, Fluvastatin, Pravastatin, Simvastatin | Statins lower cardiovascular risk, improving quality of life and reducing risk of depression | | | | • |  |  |
| Immunological effects | | | | | | |  |  |  |
| Ait Tayeb 2020 | Human | Rosuvastatin | SOD2 polymorphism associated with statin response is not associated with antidepressant or anti-inflammatory response | | | | • |  |  |
| Hai-Na 2020 | Animal | Atorvastatin | Atorvastatin reduces depressive-like symptoms by reducing pro-inflammatory cytokines and microglia activation | | | | • |  |  |
| Kang 2016 | Human | Atorvastatin, Fluvastatin, Lovastatin, Mevastatin, Pravastatin, Rosuvastatin | Statins compensate the pro-inflammatory effects of IL18 and IL6, with antidepressant effects | | | | • |  |  |
| Kim 2018 | Human | NR | Statins compensate the pro-inflammatory effects of IL18 and IL6, with antidepressant effects | | | | • |  |  |
| Lesperance 2004 | Human | NR | Statins compensate the pro-inflammatory effects of CRP | | | | • |  |  |
| Lim 2017 | Animal | Simvastatin | Simvastatin exerts antidepressant-like effects via reduction of neuronal apoptosis, microglia, and TNF-α expression | | | | • |  |  |
| Ma 2016 | Human | Atorvastatin | Atorvastatin antidepressant effects are mediated by downregulation of IL1β | | | | • |  |  |
| Menze 2021 | Animal | Simvastatin | Simvastatin exerts antidepressant-like effects via inhibition of NLRP inflammasome | | | | • |  |  |
| Neurauter 2003 | In vitro | Atorvastatin | Atorvastatin inhibits IFN-γ-mediated pathways, downregulating T cells and macrophages | | | | • |  |  |
| Wirleitner 2003 | Human | NR | Coronary heart disease is associated with decreased tryptophan levels | | | | • |  |  |
| Wu 2019 | Animal | Simvastatin | Simvastatin reduce expression of pro-inflammatory cytokines in the hippocampus | | | | • |  |  |
| Yan 2020 | Animal | Simvastatin | | Simvastatin shows antidepressant-like effects through modulation of NMDA receptor activity and inhibition of expression of COX2 and TNF-alpha | | | • |  |  |
| Yu 2019 | Animal | Simvastatin | Simvastatin exerts antidepressant-like effects via modulation of NF-κB activity | | | | • |  |  |
| Zhang 2017 | Animal | Simvastatin | Simvastatin induces depressive-like symptoms via neuroinflammation | | | | • |  |  |
| Other or non-specified | | | | | | |  |  |  |
| Gudadappanavar 2013 | Animal | Simvastatin, Lovastatin, Atorvastatin | Statins have no antidepressant-like effects | | | | • |  |  |
| Iqubal 2015 | Animal | Pitavastatin | No antidepressant-like effects of pitavastatin | | | | • |  |  |
| Jyothsna 2018 | Animal | Atorvastatin, Pitavastatin, Rosuvastatin | Statins have anxiolytic-like properties | | | | • |  |  |
| Kilic 2010 | Animal | Simvastatin, Lovastatin | Lipophilic statins have antidepressant-like effects | | | | • |  |  |

## S3 – List of clinical studies

|  | **Positive effect** | **No effect** | **Negative effect** |
| --- | --- | --- | --- |
| *Meta-analyses of randomised controlled trials* | Bai 2020 | Husain 2023 |  |
|  | De Giorgi 2021 | De Giorgi 2022 |  |
|  | Köhler-Forsberg 2019 | Hang 2021 |  |
|  | Lee 2021 | O’Neil 2012 |  |
|  | Salagre 2016 |  |  |
| *Clinical trials, randomised* | Gengo 1995 | Berk 2020 | Hyyppä 2003 |
|  | Ghanizadeh 2013 | Carlsson 2002 |  |
|  | Gougol 2015 | Chan 2017 |  |
|  | Haghighi 2014 | Harrison 1994 |  |
|  | Sparks 2005 | Fotso Soh 2020 |  |
|  |  | Massardo 2022 |  |
|  |  | Morales 2006 |  |
|  |  | Muldoon 2000 |  |
|  |  | Robertson 2017 |  |
|  |  | Santanello 1997 |  |
|  |  | Sheridan 2014 |  |
|  |  | Stewart 2000 |  |
|  |  | Visseren 2001 |  |
|  |  | Wardle 1996 |  |
| *Clinical trials, non-randomised* | Krysiak 2018 |  |  |
|  | Ormiston 2003 |  |  |
| *Meta-analyses of observational studies* | Parsaik 2014 | Lee 2021 |  |
|  |  | Zhang 2022 |  |
| *Observational studies, cohort* | Chuang 2014 | Al Badarin 2013 | Kang 2015 |
|  | Kang 2016 | Asplund 2011 | Köhler-Forsberg 2019 |
|  | Katzan 2017 | Feng 2010 | Li 2021 |
|  | Kessing 2019 | Glaus 2015 |  |
|  | Khokhar 2018 | Hoogwegt 2013 |  |
|  | Kim 2014 | Huang 2017 |  |
|  | Kim 2015 | Mansi 2013 |  |
|  | Kim 2018 | Medici 2017 |  |
|  | Köhler 2016 | Smeeth 2009 |  |
|  | Molero 2020 | Wee 2016 |  |
|  | Otte 2012 | Williams 2016 |  |
|  | Pasco 2010 |  |  |
|  | Redlich 2014 |  |  |
|  | Stafford 2011 |  |  |
|  | Wang 2017 |  |  |
|  | Wium-Andersen 2017a |  |  |
|  | Wium-Andersen 2017b |  |  |
|  | Yeh 2019 |  |  |
|  | Young-Xu 2003 |  |  |
| *Observational studies, case-control* | Williams 2016 | Pasco 2010 |  |
|  | Yang 2003 |  |  |
| *Observational studies, cross-sectional* | Otte 2012 | Agustini 2019 | Alghamdi 2018 |
|  | Feng 2008 | Lindberg 1998 | Asuzu 2015 |
|  | Mandas 2014 | Williams 2015 | Boumendil 1995 |
|  |  | Agostini 2007 | Leutner 2021 |
|  |  | Olson 2008 |  |
| *Case series* |  |  | Cham 2016 |
|  |  |  | Davidson 1996 |
|  |  |  | Duits 1993 |
|  |  |  | Lechleitner 1992 |
|  |  |  | Rosenson 1993 |
|  |  |  | Tatley 2007 |

*References*

Agostini, J. V., Tinetti, M. E., Han, L., McAvay, G., Foody, J. M., & Concato, J. (2007). Effects of Statin Use on Muscle Strength, Cognition, and Depressive Symptoms in Older Adults. *Journal of the American Geriatrics Society*, *55*(3), 420-425. <https://doi.org/10.1111/j.1532-5415.2007.01071.x>

Agustini, B., Mohebbi, M., Woods, R. L., McNeil, J. J., Nelson, M. R., Shah, R. C., Murray, A. M., Ernst, M. E., Reid, C. M., Tonkin, A., Lockery, J. E., & Berk, M. (2019). Association Between Statin Use and Depressive Symptoms in a Large Community-Dwelling Older Population Living in Australia and the USA: A Cross-Sectional Study. *CNS Drugs*, *33(7)*, 685-694. <https://ovidsp.ovid.com/ovidweb.cgi?T=JS&CSC=Y&NEWS=N&PAGE=fulltext&D=emed20&AN=627607913>

Al Badarin, F. J., Spertus, J. A., Gosch, K. L., Buchanan, D. M., & Chan, P. S. (2013). Initiation of statin therapy after acute myocardial infarction is not associated with worsening depressive symptoms: Insights from the Prospective Registry Evaluating Outcomes After Myocardial Infarctions: Events and Recovery (PREMIER) and Translational R. *American Heart Journal*, *166*(5), 879-886. <https://doi.org/10.1016/J.AHJ.2013.09.001>

Alghamdi, J., Matou-Nasri, S., Alghamdi, F., Alghamdi, S., Alfadhel, M., & Padmanabhan, S. (2018). Risk of Neuropsychiatric Adverse Effects of Lipid-Lowering Drugs: A Mendelian Randomization Study. *Int J Neuropsychopharmacol*, *21*(12), 1067-1075. <https://doi.org/10.1093/ijnp/pyy060>

Asplund, K., & Eriksson, M. (2011). Inflammation, poststroke depression and statins. *International Journal of Stroke*, *6*(6), 567-568. <https://doi.org/10.1111/j.1747-4949.2011.00691.x>

Asuzu, K., Aneni, E., Esposito, A. P., Oni, E., Veledar, E., Feldman, T., Agatston, A. S., Freitas, W., Santos, R. D., Quaglia, L. A., Guariento, M. E., Sposito, A., & Nasir, K. (2015). Statin use is associated with prevalent depression in asymptomatic octogenarians. *Circulation*, *131*(SUPPL. 1). <http://ovidsp.ovid.com/ovidweb.cgi?T=JS&PAGE=reference&D=emed16&NEWS=N&AN=71819629> (American Heart Association's Epidemiology and Prevention/Lifestyle and Cardiometabolic Health 2015 Scientific Sessions. Baltimore, MD United States.)

Bai, S., Guo, W., Feng, Y., Deng, H., Li, G., Nie, H., Guo, G., Yu, H., Ma, Y., Wang, J., Chen, S., Jing, J., Yang, J., Tang, Y., & Tang, Z. (2020). Efficacy and safety of anti-inflammatory agents for the treatment of major depressive disorder: a systematic review and meta-analysis of randomised controlled trials. *J Neurol Neurosurg Psychiatry*, *91*(1), 21-32. <https://doi.org/10.1136/jnnp-2019-320912>

Berk, M., Mohebbi, M., Dean, O. M., Cotton, S. M., Chanen, A. M., Dodd, S., Ratheesh, A., Amminger, G. P., Phelan, M., Weller, A., Mackinnon, A., Giorlando, F., Baird, S., Incerti, L., Brodie, R. E., Ferguson, N. O., Rice, S., Schäfer, M. R., Mullen, E., . . . Davey, C. G. (2020). Youth Depression Alleviation with Anti-inflammatory Agents (YoDA-A): a randomised clinical trial of rosuvastatin and aspirin. *BMC Med*, *18*(1), 16. <https://doi.org/10.1186/s12916-019-1475-6>

Boumendil, E., & Tubert-Bitter, P. (1995). Depression-induced absenteeism in relation to antihyperlipidemic treatment: A study using GAZEL cohort data. *Epidemiology*, *6(3)*, 322-325. <https://ovidsp.ovid.com/ovidweb.cgi?T=JS&CSC=Y&NEWS=N&PAGE=fulltext&D=emed5&AN=25133920>

Carlsson, C. M., Papcke-Benson, K., Carnes, M., McBride, P. E., & Stein, J. H. (2002). Health-related quality of life and long-term therapy with pravastatin and tocopherol (vitamin E) in older adults. *Drugs and Aging*, *19(10)*, 793-805. <https://ovidsp.ovid.com/ovidweb.cgi?T=JS&CSC=Y&NEWS=N&PAGE=fulltext&D=emed7&AN=35305838>

Cham, S., Koslik, H. J., & Golomb, B. A. (2016). Mood, Personality, and Behavior Changes During Treatment with Statins: A Case Series. *Drug Safety - Case Reports*, *3*(1), 1-1. <https://doi.org/10.1007/s40800-015-0024-2>

Chan, D., Binks, S., Nicholas, J. M., Frost, C., Cardoso, M. J., Ourselin, S., Wilkie, D., Nicholas, R., & Chataway, J. (2017). Effect of high-dose simvastatin on cognitive, neuropsychiatric, and health-related quality-of-life measures in secondary progressive multiple sclerosis: secondary analyses from the MS-STAT randomised, placebo-controlled trial. *Lancet Neurology*, *16*(8), 591-600. <https://ovidsp.ovid.com/ovidweb.cgi?T=JS&CSC=Y&NEWS=N&PAGE=fulltext&D=med14&AN=28600189>

Chuang, C. S., Yang, T. Y., Muo, C. H., Su, H. L., Sung, F. C., & Kao, C. H. (2014). Hyperlipidemia, statin use and the risk of developing depression: a nationwide retrospective cohort study. *Gen Hosp Psychiatry*, *36*(5), 497-501. <https://doi.org/10.1016/j.genhosppsych.2014.05.008>

Davidson, K. W., Reddy, S., McGrath, P., Zitner, D., & MacKeen, W. (1996). Increases in depression after cholesterol-lowering drug treatment [Medical Treatment of Physical Illness 3363]. *Behavioral Medicine*, *22*(2), 82-84. <https://doi.org/https://dx.doi.org/10.1080/08964289.1996.9933768> (Journal of Human Stress)

De Giorgi, R., De Crescenzo, F., Rizzo Pesci, N., Martens, M., Howard, W., Cowen, P. J., & Harmer, C. J. (2021). Statins for major depressive disorder: A systematic review and meta-analysis of randomized controlled trials. *PLoS One*, *16*(3), e0249409. <https://doi.org/10.1371/journal.pone.0249409>

De Giorgi R, Waters S, Pesci NR, Rosso G, Cowen PJ, Harmer CJ (2022). The effects of statin monotherapy on depressive symptoms: A systematic review and meta-analysis. *J Affect Disord. 2022 Aug 15;311:336-343*. https//doi.org/10.1016/j.jad.2022.05.113

Duits, N., & Bos, F. M. (1993). Depressive symptoms and cholesterol-lowering drugs. *The Lancet*, *341*(8837), 114-114. <https://doi.org/10.1016/0140-6736(93)92591-G>

Feng, L., Tan, C.-H., Merchant, R.A., Ng, T.-P. (2008). Association between Depressive Symptoms and Use of HMG-CoA Reductase Inhibitors (Statins), Corticosteroids and Histamine H2 Receptor Antagonists in Community-Dwelling Older Persons. *Drugs Aging 25, 795–805.* https://doi.org/10.2165/00002512-200825090-00005

Feng, L., Yap, K. B., Kua, E. H., & Ng, T. P. (2010). Statin use and depressive symptoms in a prospective study of community-living older persons. *Pharmacoepidemiol Drug Saf*, *19*(9), 942-948. <https://doi.org/10.1002/pds.1993>

Fotso Soh, J., Almadani, A., Beaulieu, S., Rajji, T., Mulsant, B. H., Su, C. L., Renaud, S., Mucsi, I., Torres-Platas, S. G., Levinson, A., Schaffer, A., Dols, A., Cervantes, P., Low, N., Herrmann, N., Mantere, O., & Rej, S. (2020). The effect of atorvastatin on cognition and mood in bipolar disorder and unipolar depression patients: A secondary analysis of a randomized controlled trial. *Journal of Affective Disorders*, *262*, 149-154. <https://ovidsp.ovid.com/ovidweb.cgi?T=JS&CSC=Y&NEWS=N&PAGE=fulltext&D=emed21&AN=2003760415>

Gengo, F., Cwudzinski, D., Kinkel, P., Block, G., Stauffer, L., & Lines, C. (1995). Effects of treatment with lovastatin and pravastatin on daytime cognitive performance. *Clinical Cardiology*, *18(4)*, 209-214. <https://ovidsp.ovid.com/ovidweb.cgi?T=JS&CSC=Y&NEWS=N&PAGE=fulltext&D=emed5&AN=25103738>

Ghanizadeh, A., & Hedayati, A. (2013). Augmentation of fluoxetine with lovastatin for treating major depressive disorder, a randomized double-blind placebo controlled-clinical trial. *Depress Anxiety*, *30*(11), 1084-1088. <https://doi.org/10.1002/da.22195>

Glaus, J., Vandeleur, C. L., Lasserre, A. M., Strippoli, M.-P. P. F., Castelao, E., Gholam-Rezaee, M., Waeber, G., Aubry, J.-M. M., Vollenweider, P., & Preisig, M. (2015). Aspirin and statin use and the subsequent development of depression in men and women: Results from a longitudinal population-based study. *Journal of Affective Disorders*, *182*, 126-131. <https://doi.org/10.1016/j.jad.2015.03.044>

Gougol, A., Zareh-Mohammadi, N., Raheb, S., Farokhnia, M., Salimi, S., Iranpour, N., Yekehtaz, H., & Akhondzadeh, S. (2015). Simvastatin as an adjuvant therapy to fluoxetine in patients with moderate to severe major depression: A double-blind placebo-controlled trial. *J Psychopharmacol*, *29*(5), 575-581. <https://doi.org/10.1177/0269881115578160>

Haghighi, M., Khodakarami, S., Jahangard, L., Ahmadpanah, M., Bajoghli, H., Holsboer-Trachsler, E., & Brand, S. (2014). In a randomized, double-blind clinical trial, adjuvant atorvastatin improved symptoms of depression and blood lipid values in patients suffering from severe major depressive disorder. *J Psychiatr Res*, *58*, 109-114. <https://doi.org/10.1016/j.jpsychires.2014.07.018>

Hang, X., Li, J., Li, Z., Zhang, Y., Ye, X., Tang, Q., & Sun, W. (2021). Comparative Efficacy and Acceptability of Anti-inflammatory Agents on Major Depressive Disorder: A Network Meta-Analysis. *Frontiers in Pharmacology*, *12*, Article 691200. <https://ovidsp.ovid.com/ovidweb.cgi?T=JS&CSC=Y&NEWS=N&PAGE=fulltext&D=emexa&AN=635485881>

Harrison, R. W. S., & Ashton, C. H. (1994). Do cholesterol-lowering agents affect brain activity? A comparison of simvastatin, pravastatin, and placebo in healthy volunteers. *British Journal of Clinical Pharmacology*, *37(3)*, 231-236. <https://ovidsp.ovid.com/ovidweb.cgi?T=JS&CSC=Y&NEWS=N&PAGE=fulltext&D=emed5&AN=24212181>

Hoogwegt, M. T., Theuns, D. A. M. J., Kupper, N., Jordaens, L., & Pedersen, S. S. (2013). Relation of Statin Therapy to Psychological Functioning in Patients With an Implantable Cardioverter Defibrillator. *The American Journal of Cardiology*, *111*(8), 1169-1174. <https://doi.org/10.1016/J.AMJCARD.2012.12.047>

Huang, C. I., Lin, L. C., Tien, H. C., Que, J., Ting, W. C., Chen, P. C., Wu, H. M., Ho, C. H., Wang, J. J., Wang, R. H., & Yang, C. C. (2017). Hyperlipidemia and statins use for the risk of new-onset anxiety/depression in patients with head and neck cancer: A population-based study. *PLoS One*, *12*(3). <https://doi.org/10.1371/journal.pone.0174574>

Husain MI, Chaudhry IB, Khoso AB, Kiran T, Khan N, Ahmad F, Hodsoll J, Husain MO, Naqvi HA, Nizami AT, Chaudhry N, Khan HA, Minhas F, Meyer JH, Ansari MA, Mulsant BH, Husain N, Young AH (2023). Effect of Adjunctive Simvastatin on Depressive Symptoms Among Adults With Treatment-Resistant Depression: A Randomized Clinical Trial. *JAMA Netw Open.* *2023 Feb 1;6(2):e230147.* https://doi.org/ 10.1001/jamanetworkopen.2023.0147

Hyyppä, M. T., Kronholm, E., Virtanen, A., Leino, A., & Jula, A. (2003). Does simvastatin affect mood and steroid hormone levels in hypercholesterolemic men? A randomized double-blind trial. *Psychoneuroendocrinology*, *28(2)*, 181-194. <https://ovidsp.ovid.com/ovidweb.cgi?T=JS&CSC=Y&NEWS=N&PAGE=fulltext&D=emed8&AN=36044077>

Kang, H. J., Bae, K. Y., Kim, S. W., Kim, J. T., Park, M. S., Cho, K. H., & Kim, J. M. (2016). Effects of interleukin-6, interleukin-18, and statin use, evaluated at acute stroke, on post-stroke depression during 1-year follow-up. *Psychoneuroendocrinology*, *72*, 156-160. <https://doi.org/10.1016/j.psyneuen.2016.07.001>

Kang, J.-H., Kao, L.-T., Lin, H.-C., Tsai, M.-C., & Chung, S.-D. (2015). Statin use increases the risk of depressive disorder in stroke patients: A population-based study. *Journal of the Neurological Sciences*, *348*(1-2), 89-93. <https://doi.org/10.1016/J.JNS.2014.11.013>

Katzan, I., Thompson, N., & Itrat, A. (2017). High-sensitivity C-reactive protein and depression in patients with ischemic stroke. *Stroke*, *48*(Supplement 1). <http://ovidsp.ovid.com/ovidweb.cgi?T=JS&PAGE=reference&D=emed18&NEWS=N&AN=617461553> (American Heart Association/American Stroke Association 2017 International Stroke Conference and State-of-the-Science Stroke Nursing Symposium. Houston, TX United States.)

Kessing, L. V., Rytgaard, H. C., Gerds, T. A., Berk, M., Ekstrøm, C. T., & Andersen, P. K. (2019). New drug candidates for depression - a nationwide population-based study. *Acta Psychiatr Scand*, *139*(1), 68-77. <https://doi.org/10.1111/acps.12957>

Khokhar, B. R., Simoni-Wastila, L., Slejko, J. F., Perfetto, E., Zhan, M., & Smith, G. S. (2018). Mortality and secondary injury following traumatic brain injury in older medicare stat in users. *Pharmacoepidemiology and Drug Safety*, *26(Supplement 2)*, 161-162. <https://ovidsp.ovid.com/ovidweb.cgi?T=JS&CSC=Y&NEWS=N&PAGE=fulltext&D=emed18&AN=618125390>

Kim, J. M., Stewart, R., Kang, H. J., Bae, K. Y., Kim, S. W., Shin, I. S., Kim, J. T., Park, M. S., Cho, K. H., & Yoon, J. S. (2014). A prospective study of statin use and poststroke depression. *J Clin Psychopharmacol*, *34*(1), 72-79. <https://doi.org/10.1097/jcp.0000000000000051>

Kim, S.-W., Kang, H.-J., Bae, K.-Y., Shin, I.-S., Hong, Y. J., Ahn, Y.-K., Jeong, M. H., Berk, M., Yoon, J.-S., & Kim, J.-M. (2018). Interactions between pro-inflammatory cytokines and statins on depression in patients with acute coronary syndrome. In (Vol. 80, pp. 250-254): Elsevier.

Kim, S. W., Bae, K. Y., Kim, J. M., Shin, I. S., Hong, Y. J., Ahn, Y., Jeong, M. H., Berk, M., & Yoon, J. S. (2015). The use of statins for the treatment of depression in patients with acute coronary syndrome. In (Vol. 5, pp. e620): Nature Publishing Group.

Köhler, O., Gasse, C., Petersen, L., Ingstrup, K. G., Nierenberg, A. A., Mors, O., & Østergaard, S. D. (2016). The Effect of Concomitant Treatment With SSRIs and Statins: A Population-Based Study. *Am J Psychiatry*, *173*(8), 807-815. <https://doi.org/10.1176/appi.ajp.2016.15040463>

Köhler-Forsberg, O., C, N. L., Hjorthøj, C., Nordentoft, M., Mors, O., & Benros, M. E. (2019). Efficacy of anti-inflammatory treatment on major depressive disorder or depressive symptoms: meta-analysis of clinical trials. *Acta Psychiatr Scand*, *139*(5), 404-419. <https://doi.org/10.1111/acps.13016>

Köhler-Forsberg, O., Gasse, C., Petersen, L., Nierenberg, A. A., Mors, O., & Østergaard, S. D. (2019). Statin treatment and the risk of depression. *J Affect Disord*, *246*, 706-715. <https://doi.org/10.1016/j.jad.2018.12.110>

Krysiak, R., Drosdzol-Cop, A., Skrzypulec-Plinta, V., & Okopień, B. (2018). The effect of atorvastatin on sexual function and depressive symptoms in young women with elevated cholesterol levels — a pilot study. *Endokrynologia Polska*, *69*(6), 688-694. <https://doi.org/10.5603/EP.a2018.0062>

Lechleitner, M., Hoppichler, F., Konwalinka, G., Patsch, J., & Braunsteiner, H. (1992). Depressive symptoms in hypercholesterolaemic patients treated with pravastatin. *The Lancet*, *340*(8824), 910-910. <https://doi.org/10.1016/0140-6736(92)93318-H>

Lee, M. C., Peng, T. R., Chen, B. L., Lee, C. H., Wang, J. Y., Lai, C. P., Lee, J. A., Chen, S. M., & Shiang, J. C. (2021). Effects of various statins on depressive symptoms: A network meta-analysis. *J Affect Disord*, *293*, 205-213. <https://doi.org/10.1016/j.jad.2021.06.034>

Lee, M. C., Peng, T. R., Lee, C. H., Wang, J. Y., Lee, J. A., Chen, S. M., & Shiang, J. C. (2021). Statin use and depression risk: A systematic review and meta-analysis. *J Affect Disord*, *282*, 308-315. <https://doi.org/10.1016/j.jad.2020.12.164>

Leutner, M., Matzhold, C., Kautzky, A., Kaleta, M., Thurner, S., Klimek, P., & Kautzky-Willer, A. (2021). Major Depressive Disorder (MDD) and Antidepressant Medication Are Overrepresented in High-Dose Statin Treatment. *Front Med (Lausanne)*, *8*, 608083. <https://doi.org/10.3389/fmed.2021.608083>

Li, Y., Guo, Y., Zhou, M., Ma, M., Fang, J., & He, L. (2021). Paradoxical effect of statin medication on depressive disorder in first-ever ischemic stroke patients: possible antidepressant-like effect prestroke and the opposite in continuous medication poststroke. *Int Clin Psychopharmacol*, *36*(3), 147-153. <https://doi.org/10.1097/yic.0000000000000352>

Lindberg, G., & Hallas, J. (1998). Cholesterol-lowering drugs and antidepressants - A study of prescription symmetry. *Pharmacoepidemiology and Drug Safety*, *7*(6), 399-402. <https://doi.org/http://dx.doi.org/10.1002/%28SICI%291099-1557%28199811/12%297:6%3C399::AID-PDS385%3E3.0.CO;2-C>

Mandas, A., Congiu, M. G., Abete, C., Dessì, S., Manconi, P. E., Musio, M., Columbu, S., & Racugno, W. (2014). Cognitive decline and depressive symptoms in late-life are associated with statin use: evidence from a population-based study of Sardinian old people living in their own home. *Neurological Research*, *36*(3), 247-254. <https://doi.org/10.1179/1743132813Y.0000000287>

Mansi, I., Frei, C. R., Pugh, M. J., & Mortensen, E. M. (2013). Psychologic disorders and statin use: a propensity score-matched analysis. *Pharmacotherapy*, *33*(6), 615-626. <https://doi.org/10.1002/phar.1272>

Massardo, T., Quintana, J. C., Risco, L., Corral, S., Spuler, J., Vicentini, D., Castro-Muñoz, G., Riedel, B., Villa, C., & Pereira, J. I. (2022). Effect of Low-Dose Statins in Addition to Standard Therapy on Brain Perfusion and Neurocognitive Performance in Patients with Major Depressive Disorder. *Neuropsychobiology*, 1-15. <https://doi.org/10.1159/000521104>

Medici, C. R., Gradus, J. L., Pedersen, L., Sorensen, H. T., Ostergaard So, D., & Christiansen, C. F. (2017). No impact of preadmission anti-inflammatory drug use on risk of depression and anxiety after critical illness. *Critical Care Medicine*, *45*(10), 1635-1641. <https://doi.org/http://dx.doi.org/10.1097/CCM.0000000000002571>

Molero, Y., Cipriani, A., Larsson, H., Lichtenstein, P., D'Onofrio, B. M., & Fazel, S. (2020). Associations between statin use and suicidality, depression, anxiety, and seizures: a Swedish total-population cohort study. *Lancet Psychiatry*, *7*(11), 982-990. <https://doi.org/10.1016/s2215-0366(20)30311-4>

Morales, K., Wittink, M., Datto, C., Difilippo, S., Cary, M., Tenhave, T., & Katz, I. R. (2006). Simvastatin causes changes in affective processes in elderly volunteers. *Journal of the American Geriatrics Society*, *54(1)*, 70-76. <https://ovidsp.ovid.com/ovidweb.cgi?T=JS&CSC=Y&NEWS=N&PAGE=fulltext&D=emed9&AN=43437374>

Muldoon, M. F., Barger, S. D., Ryan, C. M., Flory, J. D., Lehoczky, J. P., Matthews, K. A., & Manuck, S. B. (2000). Effects of lovastatin on cognitive function and psychological well-being. *American Journal of Medicine*, *108(7)*, 538-546. <https://ovidsp.ovid.com/ovidweb.cgi?T=JS&CSC=Y&NEWS=N&PAGE=fulltext&D=emed7&AN=30249031>

O'Neil, A., Sanna, L., Redlich, C., Sanderson, K., Jacka, F., Williams, L. J., Pasco, J. A., & Berk, M. (2012). The impact of statins on psychological wellbeing: 12A systematic review and meta-analysis. *BMC Medicine*, *10*, 154. <https://doi.org/http://dx.doi.org/10.1186/1741-7015-10-154>

Olson, M. B., Kelsey, S. F., Matthews, K. A., Bairey Merz, C. N., Eteiba, W., McGorray, S. P., Cornell, C. E., Vido, D. A., & Muldoon, M. F. (2008). Lipid-lowering medication use and aggression scores in women: a report from the NHLBI-sponsored WISE study. *Journal of women's health (2002)*, *17*(2), 187-194. <https://doi.org/10.1089/jwh.2007.0379>

Ormiston, T., Wolkowitz, O. M., Reus, V. I., & Manfredi, F. (2003). Behavioral implications of lowering cholesterol levels: a double-blind pilot study. *Psychosomatics*, *44*(5), 412-414. <https://ovidsp.ovid.com/ovidweb.cgi?T=JS&CSC=Y&NEWS=N&PAGE=fulltext&D=med5&AN=12954916>

Otte, C., Zhao, S., & Whooley, M. A. (2012). Statin Use and Risk of Depression in Patients With Coronary Heart Disease. *The Journal of Clinical Psychiatry*, *73*(05), 610-615. <https://doi.org/10.4088/JCP.11m07038>

Pasco, J. A., Jacka, F. N., Williams, L. J., Henry, M. J., Nicholson, G. C., Kotowicz, M. A., & Berk, M. (2010). Clinical Implications of the Cytokine Hypothesis of Depression: The Association between Use of Statins and Aspirin and the Risk of Major Depression. *Psychotherapy and Psychosomatics*, *79*(5), 323-325. <https://doi.org/10.1159/000319530>

Redlich, C., Berk, M., Williams, L. J., Sundquist, J., Sundquist, K., & Li, X. (2014). Statin use and risk of depression: a Swedish national cohort study. *BMC Psychiatry*, *14*, 348. <https://doi.org/10.1186/s12888-014-0348-y>

Robertson, C. S., McCarthy, J. J., Miller, E. R., Levin, H., McCauley, S. R., & Swank, P. R. (2017). Phase II Clinical Trial of Atorvastatin in Mild Traumatic Brain Injury. *Journal of Neurotrauma*, *34(7)*, 1394-1401. <https://ovidsp.ovid.com/ovidweb.cgi?T=JS&CSC=Y&NEWS=N&PAGE=fulltext&D=emed18&AN=615273914>

Rosenson, R. S., & Goranson, N. L. (1993). Lovastatin-associated sleep and mood disturbances. *The American journal of medicine*, *95*(5), 548-549. <https://doi.org/10.1016/0002-9343(93)90343-n>

Salagre, E., Fernandes, B. S., Dodd, S., Brownstein, D. J., & Berk, M. (2016). Statins for the treatment of depression: A meta-analysis of randomized, double-blind, placebo-controlled trials. *J Affect Disord*, *200*, 235-242. <https://doi.org/10.1016/j.jad.2016.04.047>

Santanello, N. C., Barber, B. L., Applegate, W. B., Elam, J., Curtis, C., Hunninghake, D. B., & Gordon, D. J. (1997). Effect of pharmacologic lipid lowering on health-related quality of life in older persons: Results from the Cholesterol Reduction in Seniors Program (CRISP) pilot study. *Journal of the American Geriatrics Society*, *45(1)*, 8-14.

Sheridan, D. A., Bridge, S. H., Crossey, M. M. E., Felmlee, D. J., Thomas, H. C., Neely, R. D. G., Taylor-Robinson, S. D., & Bassendine, M. F. (2014). Depressive symptoms in chronic hepatitis C are associated with plasma apolipoprotein E deficiency [Journal: Article]. *Metabolic brain disease*, *29*(3), 625‐634. <https://doi.org/10.1007/s11011-014-9520-9>

Smeeth, L., Douglas, I., Hall, A. J., Hubbard, R., & Evans, S. (2009). Effect of statins on a wide range of health outcomes: a cohort study validated by comparison with randomized trials. *British Journal of Clinical Pharmacology*, *67*(1), 99-109. <https://doi.org/10.1111/j.1365-2125.2008.03308.x>

Sparks, D. L., Sabbagh, M. N., Connor, D. J., Lopez, J., Launer, L. J., Browne, P., Wasser, D., Johnson-Traver, S., Lochhead, J., & Ziolwolski, C. (2005). Atorvastatin for the treatment of mild to moderate Alzheimer disease: Preliminary results. *Archives of Neurology*, *62(5)*, 753-757. <https://ovidsp.ovid.com/ovidweb.cgi?T=JS&CSC=Y&NEWS=N&PAGE=fulltext&D=emed9&AN=40656090>

Stafford, L., & Berk, M. (2011). The Use of Statins After a Cardiac Intervention Is Associated With Reduced Risk of Subsequent Depression. *The Journal of Clinical Psychiatry*, *72*(09), 1229-1235. <https://doi.org/10.4088/JCP.09m05825blu>

Stewart, R. A., Sharples, K. J., North, F. M., Menkes, D. B., Baker, J., & Simes, J. (2000). Long-term assessment of psychological well-being in a randomized placebo-controlled trial of cholesterol reduction with pravastatin. *Archives of Internal Medicine*, *160(20)*, 3144-3152. <https://ovidsp.ovid.com/ovidweb.cgi?T=JS&CSC=Y&NEWS=N&PAGE=fulltext&D=emed7&AN=30823456>

Tatley, M., & Savage, R. (2007). Psychiatric Adverse Reactions with Statins, Fibrates and Ezetimibe. *Drug Safety*, *30*(3), 195-201. <https://doi.org/10.2165/00002018-200730030-00003>

Visseren, F. L. J., Bouter, P. K., Van Loon, B. J. P., & Erkelens, W. D. (2001). Treatment of dyslipidaemia with fluvastatin in patients with type 2 diabetes mellitus: Effects on lipids, mental state and fibrinolysis. *Clinical Drug Investigation*, *21(10)*, 671-678. <https://ovidsp.ovid.com/ovidweb.cgi?T=JS&CSC=Y&NEWS=N&PAGE=fulltext&D=emed7&AN=33027826>

Wang, Z., Lam, B., Wong, A., Xiong, Y., & Mok, V. (2017). Predictors for post-stroke depression-three-year longitudinal study. *Cerebrovascular Diseases*, *44*(Supplement 1), 47. <https://doi.org/http://dx.doi.org/10.1159/000481589> (Annual Conference of the Asia Pacific Stroke Organization Combined with Stroke Society of Australasia, APSO 2017. Nanjing China.)

Wardle, J., Armitage, J., Collins, R., Wallendszus, K., Keech, A., & Lawson, A. (1996). Randomised placebo controlled trial of effect on mood of lowering cholesterol concentration. *British Medical Journal*, *313(7049)*, 75-78. <https://ovidsp.ovid.com/ovidweb.cgi?T=JS&CSC=Y&NEWS=N&PAGE=fulltext&D=emed6&AN=26257756>

Wee, H.-Y., Ho, C.-H., Fu, Liang, W., Hsieh, K.-Y., Wang, C.-C., Wang, J.-J., Chio, C.-C., Chang, C.-H., & Kuo, J.-R. (2016). Increased Risk of New-Onset Depression in Patients With Traumatic Brain Injury and Hyperlipidemia: The Important Role of Statin Medications. *J Clin Psychiatry*, *77*(4), 505-511. <https://doi.org/10.4088/JCP.14m09749>

Williams, E. D., Eastwood, S. V., Tillin, T., Stewart, R., Chaturvedi, N., & Hughes, A. D. (2015). Statin use is associated with reduced depressive symptoms in Europeans, but increased symptoms in ethnic minorities in the UK: an observational study. *British Journal of Clinical Pharmacology*, *80*(1), 172-173. <https://doi.org/10.1111/bcp.12599>

Williams, L. J., Pasco, J. A., Mohebbi, M., Jacka, F. N., Stuart, A. L., Venugopal, K., O'Neil, A., & Berk, M. (2016). Statin and Aspirin Use and the Risk of Mood Disorders among Men. *The international journal of neuropsychopharmacology*, *19*(6). <https://doi.org/10.1093/ijnp/pyw008>

Wium-Andersen, I. K., Wium-Andersen, M. K., Jørgensen, M. B., & Osler, M. (2017a). Anti-inflammatory treatment and risk for depression after first-time stroke in a cohort of 147 487 Danish patients. *J Psychiatry Neurosci*, *42*(5), 320-330. <https://doi.org/10.1503/jpn160244>

Wium-Andersen, I. K., Wium-Andersen, M. K., Jørgensen, M. B., & Osler, M. (2017b). Anti-inflammatory treatment and risk of depression in 91,842 patients with acute coronary syndrome and 91,860 individuals without acute coronary syndrome in Denmark. *Int J Cardiol*, *246*, 1-6. <https://doi.org/10.1016/j.ijcard.2017.05.105>

Yang, C.-C., Jick, S. S., & Jick, H. (2003). Lipid-Lowering Drugs and the Risk of Depression and Suicidal Behavior. In (Vol. 163, pp. 1926): American Medical Association.

Yeh, J.-J., Syue, S.-H., Lin, C.-L., Hsu, C. Y., Shae, Z., & Kao, C.-H. (2019). Effects of statins on anxiety and depression in patients with asthma-chronic obstructive pulmonary disease overlap syndrome. *Journal of Affective Disorders*, *253*, 277-284. <https://doi.org/10.1016/J.JAD.2019.05.002>

Young-Xu, Y., Chan, K. A., Liao, J. K., Ravid, S., & Blatt, C. M. (2003). Long-term statin use and psychological well-being. *Journal of the American College of Cardiology*, *42*(4), 690-697. <https://doi.org/10.1016/S0735-1097(03)00785-X>

Zhang, L., Bao, Y., Tao, S., Zhao, Y., & Liu, M. (2022). The association between cardiovascular drugs and depression/anxiety in patients with cardiovascular disease: A meta-analysis. *Pharmacol Res*, *175*, 106024. <https://doi.org/10.1016/j.phrs.2021.106024>

## S4 – Adapted SANRA (Scale for the quality Assessment of Narrative Review Articles)

**1) Justification of the article’s importance for the readership**

⇑ The importance is explicitly justified 🡪 see **Introduction**, page 3; Aim of the review, pages 6-7

**2) Statement of concrete aims or formulation of questions**

⇑ One or more concrete aims or questions are formulated 🡪 see Aim of the review, pages 6-7

**3) Description of the literature search**

⇑ The literature search is described in detail, including search terms and inclusion criteria 🡪 see **Materials and Methods**, pages 7-8; Supplementary Material, S1 – Search strategy

**4) Referencing**

⇑ Key statements are supported by references 🡪 see **References**, pages 21-35; throughout the text

**5) Scientific reasoning**

⇑ Appropriate evidence is generally present 🡪 see **Results**, pages 8-17 (only included mechanistic studies that reported original data on the pharmacological effects of statins in depression, with no restriction to their design and language)

**6) Appropriate presentation of data**

⇔ Relevant outcome data are generally presented appropriately 🡪 see **Results**, pages 8-17 (data are presented descriptively, as effect sizes with confidence intervals were generally not available for this kind of mechanistic research)
